# Neuronal Nitric Oxide Synthase and Human Vascular Regulation

**DOI:** 10.1016/j.tcm.2010.02.007

**Published:** 2009-11

**Authors:** Narbeh Melikian, Michael D. Seddon, Barbara Casadei, Philip J. Chowienczyk, Ajay M. Shah

**Affiliations:** a King's College London British Heart Foundation Centre of Excellence, Cardiovascular Division, London, United Kingdom; b Department of Cardiovascular Medicine, University of Oxford, Oxford, United Kingdom

## Abstract

Vascular blood flow and its distribution among different vascular beds are regulated by changes in microvascular tone. Nitric oxide (NO) plays a key role in the local paracrine regulation of vessel tone both under resting conditions and when blood flow increases in response to agonist stimulation or increased shear stress. The conventional notion that endothelial NO synthase (eNOS)-derived NO is largely responsible for both effects has been challenged by first-in-human studies with a selective inhibitor of neuronal NOS (nNOS), *S*-methyl-l-thiocitrulline (SMTC). These studies reveal that SMTC causes a reduction in basal blood flow in the normal human forearm and coronary circulations (that is reversed by l-arginine), without affecting the eNOS-mediated vasodilatation elicited by acetylcholine, substance P, or increased shear stress. *S*-methyl-l-thiocitrulline also inhibits mental stress-induced vasodilatation. These results are consistent with a significant body of experimental studies suggesting that nNOS plays an important role in the local regulation of vessel tone in other species, independent of the effects of nNOS-derived NO in the central nervous system. These emerging data suggest that eNOS and nNOS have distinct roles in the physiologic local regulation of human microvascular tone in vivo and pave the way for further detailed investigation of the relative contribution of nNOS and eNOS in vascular regulation in human disease.

## Introduction

The regulation of blood flow distribution among different vascular beds according to varying metabolic requirements is fundamentally important in ensuring a match between tissue perfusion and metabolism. This is primarily achieved through complex adjustments in the tone and thus resistance of the microcirculation. Dysregulation or impairment of microvascular function may lead to organ dysfunction in virtually any body system. At a local level, nitric oxide (NO) is well known to play a pivotal role in the modulation of microvascular tone (Moncada and Higgs 1993, 2006). Nitric oxide (NO) has potent vasodilator effects that interact with other local vasoactive mediators (eg, prostanoids, endothelin, and the endothelium-derived hyperpolarizing factor) and with neural inputs to determine overall vascular tone and blood flow (Moncada and Higgs 1993, 2006). Although three distinct isoforms of NO synthase (NOS)—endothelial NOS (eNOS), neuronal NOS (nNOS), and inducible NOS—may generate NO with the use of l-arginine as substrate, it has generally been accepted that the local regulation of vascular tone in health is primarily dependent upon the release of NO from eNOS (Deanfield et al. 2007, Moncada and Higgs 2006). However, an emerging literature from animal studies has suggested that nNOS-derived NO may also be involved in this process. These experimental findings have now been complemented by a series of recent first-in-human studies providing evidence that nNOS-derived NO plays an important role in the local regulation of basal microvascular tone as well as in the vasodilator response to mental stress.

## Endothelial NOS and acute vasodilatation

A large body of data from animal and human studies for more than two decades indicates that NO generated in the endothelium induces paracrine relaxation of vascular smooth muscle cells leading to a reduction in vascular tone (Moncada et al. 1993, 2006, Palmer et al. 1988). These NO-dependent vasodilator effects are elicited by increases in endothelial pulsatile shear stress or by a wide variety of agonists (eg, acetylcholine, substance P, and thrombin) that act on the vascular endothelium (Deanfield et al. 2007, Vallance and Chan 2001). The most compelling evidence that these effects involve eNOS comes from studies in eNOS knockout mice, where both agonist- and flow-induced NO-dependent vasorelaxation are impaired (Godecke et al. 1998, Huang et al. 1995, Scotland et al. 2002). In humans, the NO synthase inhibitor N^G^ monomethyl-l-arginine (l-NMMA) inhibits both agonist- and flow-induced vasodilatation in several vascular beds (including the forearm and coronary circulation) providing further evidence that NO is involved in the regulation of vascular tone (Drexler 1997, Moncada et al. 1993, 2006). Although l-NMMA inhibits the activity of all NOS isoforms, the observation that eNOS is the major NOS isoform expressed in endothelial cells combined with ex vivo studies in human vessels confirming the central role of the endothelium in vascular responses support the conclusion that eNOS is the predominant source of NO responsible for the regulation of vascular tone (Moncada et al. 1993, 2006). This conclusion is further supported by evidence that impairment of eNOS-dependent (agonist- and flow-induced) vasodilatation in humans (generally termed *endothelial dysfunction*) is a prognostic marker for future atherosclerosis-related cardiovascular events (Landmesser et al. 2004, Quyyumi 2003).

## Nitric oxide-dependent regulation of basal vascular tone

Experimental studies with the use of l-NMMA and other nonselective NOS inhibitors have suggested that continuous release of NO from the endothelium might tonically reduce basal microvascular tone in vivo—an effect termed *tonic vasodilatation* (Moncada et al. 1993, 2006). In healthy humans, seminal studies by Vallance et al. (Vallance et al. 1989) found that local forearm infusion of l-NMMA reduced resting blood flow. The effects of l-NMMA were abrogated by l-arginine but not d-arginine, indicating that stereospecific inhibition of the l-arginine/NO pathway was involved (Vallance et al. 1989). Similar results have been confirmed by numerous investigators, and the data extended to other vascular beds, including the coronary and pulmonary circulations (Moncada et al. 1993, 2006). Because l-NMMA reduces basal NO production by cultured endothelial cells and induces vasoconstriction in isolated vascular rings, and eNOS-derived NO is involved in agonist-induced vasodilation, it has generally been considered that sustained release of NO by eNOS is the underlying mechanism for “tonic vasodilatation” observed in vivo (Moncada et al. 1993, 2006). Further data that are often interpreted as supporting NO-dependent tonic vasodilatation are the findings from both animal and human studies that systemic infusion of l-NMMA causes acute dose-dependent hypertension (Rees et al. 1989, Haynes et al. 1995).

However, it is important to recognize that the above data do not offer definitive proof for the involvement of eNOS in regulation of vascular tone. Although l-NMMA is specific for NOS, it is nonselective for its three isoforms and, therefore, does not provide any evidence for the involvement of an individual NOS isoform. With regard to the data on l-NMMA-induced hypertension, it should be noted that both kidney function and autonomic nerve activity are influenced by NO and have a major impact on blood pressure regulation; thus, the systemic effects of NOS inhibition cannot be interpreted as merely reflecting changes in endothelial function and local vascular tone. In addition, there are incongruous results from human studies that provide a basis to challenge the notion that eNOS is the major source of NO responsible for the local regulation of microvascular tone. For example, several investigators have shown that there is often a poor correlation between basal and stimulated NO-dependent vasodilatation within the same vascular bed—with a reduction in the stimulated response but a relative preservation of the basal response (Deanfield et al. 2007, Drexler 1997). Such differences are particularly marked in the presence of cardiovascular risk factors or in individuals with established atherosclerotic vascular disease. In fact, direct in vivo evidence that a continuous eNOS-dependent local release of NO modulates basal vascular tone is lacking. Hence, the question arises whether local regulation of basal microvascular tone by NO is subserved by a different source of NO.

## Neuronal NOS and vascular tone

The nNOS isoform is expressed in most regions of the central nervous system, in parasympathetic ganglia and in selected nonadrenergic noncholinergic peripheral autonomic nerve fibers (or “nitrergic” nerves) (Schuman and Madison 1994, Toda and Okamura 2003). The nNOS-derived NO is an important neurotransmitter that is involved in neuronal plasticity (in particular in memory formation), regulation of central nervous system blood flow, peripheral and central transmission of pain signals, neurotransmitter release from cholinergic nerve fibers, and the functional regulation of organs with nitrergic innervation (Schuman and Madison 1994, Toda and Okamura 2003). In addition to the central nervous system, nNOS is now known to also be expressed in many nonneuronal cell types such as skeletal (Kobzik et al. 1994) and cardiac myocytes (Xu et al. 1999), cells of the macula densa within the kidney (Bachmann et al. 1995), and selected smooth muscle (Boulanger et al. 1998) and endothelial (Bachetti et al. 2004) cells. The availability of selective nNOS inhibitors, as well as studies in gene-modified mice with perturbation of nNOS expression, have provided evidence that nonneuronal nNOS is physiologically active and exerts important regulatory influences in a number of different tissues.

A significant body of in vitro and in vivo experimental data implicates nNOS-derived NO in local physiologic regulation of vascular tone acting through several different mechanisms. These local regulatory influences appear to be independent of any central actions of nNOS that affect autonomic function. In the renal and cerebral circulation, a number of studies have suggested that nNOS-derived NO is involved in the autoregulation of local blood flow through direct effects on vascular smooth muscle (Bauser-Heaton and Bohlen 2007, Chi et al. 2003, Hudetz et al. 1998, Ichihara et al. 1998, Mattson and Meister 2005, Pelligrino et al. 1993, Santizo et al. 2000, Sigmon and Beierwaltes 2000, Vallon et al. 2001).

With the use of the selective nNOS-inhibitor *S*-methyl-l-thiocitrulline (SMTC) in explanted whole rat kidneys, Ichihara et al. (Ichihara et al. 1998) provided the first direct evidence for nNOS-dependent regulation of vascular tone in the kidney. Selective inhibition of nNOS decreased basal afferent and efferent arteriolar tone but had no effect on vasodilatation in response to the eNOS agonist acetylcholine. These investigators further demonstrated that removal of the cells of the macula densa by papillectomy (the main source of renal nNOS) abolished the vasoconstrictor effects of SMTC (Ichihara et al. 1998). Additional direct evidence for the role of nNOS-derived NO in the kidney is derived from nNOS knockout mice (nNOS^−/−^), where the regulatory influences of the macula densa are significantly attenuated (Vallon et al. 2001).

Other studies have suggested that nNOS may be involved in the reflex regulation of cerebral vascular tone and blood flow, particularly in response to hypoxia and/or hypotension (Bauser-Heaton and Bohlen 2007, Chi et al. 2003, Hudetz et al. 1998, Pelligrino et al. 1993, Santizo et al. 2000). For example, with the use of an in vivo rat model, Bauser-Heaton et al. (Bauser-Heaton and Bohlen 2007) demonstrated that selective inhibition of nNOS with *N*-(4*S*)-(4-amino-5-[aminoethyl]aminopentyl)-*N*'-nitroguanidine reduced basal cerebral arterial diameter and abolished the vasodilator response to hypoxia. In contrast, flow-mediated dilatation (a predominant eNOS-mediated response) remained intact in the presence of selective nNOS inhibition. This study did not directly investigate the cellular sources of nNOS, which could potentially include the arteriolar smooth muscle cell or perivascular nerve fibers.

Other studies provide evidence that nNOS-derived NO may alter vascular tone through inhibition of perivascular sympathetic nerve activity. In isolated rat mesenteric arteries without endothelium, Hatanaka et al. (Hatanaka et al. 2006) showed that selective nNOS inhibition with vinyl-l-5-(1-imino-3-butenyl)-l-ornithine (l-VNIO) augmented arterial vasoconstriction and local norepinephrine concentration in response to perivascular nerve stimulation. However, l-VNIO did not influence the vasoconstrictor response to exogenous norepinephrine, suggesting that nNOS-derived NO may affect neurotransmitter release from perivascular sympathetic nerves. Consistent with this idea, immunostaining of mesenteric artery specimens localized nNOS to perivascular capsaicin-sensitive sensory nerve fibers, whereas the functional depletion of nNOS-immunopositive fibers with capsaicin abolished the augmented vasoconstrictor response to nerve stimulation (Hatanaka et al. 2006). Taken together, these observations suggest that nNOS-derived NO released from capsaicin-sensitive sensory nerve fibers may presynaptically modulate adrenergic sympathetic neurotransmission and hence vascular tone.

Skeletal muscle may be an additional source of nNOS-derived NO with important influences on vascular tone. In normal skeletal muscle, nNOS is located at the cell membrane bound to the cytoskeletal protein dystrophin (Brenman et al. 1995, Chang et al. 1996). The absence of dystrophin, as seen in patients with Duchenne muscular dystrophy (DMD) and its animal equivalent the *mdx* mouse, leads to a significant reduction in skeletal muscle nNOS expression and in skeletal muscle blood flow (Brenman et al. 1995). With the use of the *mdx* mouse model, Thomas et al. (Thomas et al. 1998) showed that the normal ability to attenuate *α*-adrenergic vasoconstriction in response to sciatic nerve stimulation in the hind limb was significantly impaired in a similar manner to that observed in nNOS-deficient mice. Furthermore, nNOS-deficient mice did not exhibit any enhancement of vasoconstriction in the contracting hind limb after treatment with *N*-nitro-l-arginine methyl ester (l-NAME) (Thomas et al. 1998). Taken together, these findings suggest that NO derived from nNOS may have an important role in maintaining blood flow in the exercising skeletal muscle by reducing *α*-adrenergic vasoconstriction and that its absence may account for the abnormal vascular responses seen in contracting limbs of *mdx* mice or nNOS knockout mice.

## In vivo human evidence for nNOS-mediated regulation of vascular tone

The main tools available to study NOS-dependent effects in humans in vivo have until recently been the nonselective NOS inhibitor l-NMMA and agonists such as acetylcholine that induce eNOS activation. The lack of isoform-selective NOS inhibitors suitable and validated for in vivo human use has been a major limitation to assessing the relative roles of individual NOS isoforms in the regulation of cardiovascular responses.

Limited circumstantial evidence for a potential role of nNOS-derived NO in the regulation of human vascular tone was initially obtained from studies in children with DMD, who are presumed to be deficient in skeletal muscle nNOS activity (Sander et al. 2000). In studies analogous to those described above for the *mdx* mouse model, Sander et al found that children with DMD had no blunting of the vasoconstrictor response (measured as a decrease in muscle oxygenation) to reflex sympathetic activation during forearm exercise (Sander et al. 2000). In contrast, the same stimulus led to reduced vasoconstriction in healthy children or in patients with different muscle disorders where nNOS expression was expected to be intact (Sander et al. 2000). Additional indirect evidence for the vascular influences of nNOS was derived from studies in renal transplant patients. Kwon et al. (Kwon et al. 2009) demonstrated that impaired graft vascular function subsequent to renal transplantation, a condition that encompasses altered renal vascular tone and loss of vascular autoregulation, was associated with diminished staining for nNOS in renal biopsy specimens.

**Figure 1:**
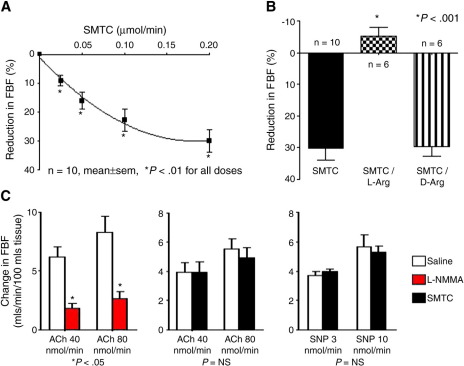
Effects of nNOS in the human forearm. Intraarterial infusion of the nNOS-specific inhibitor STMC into the brachial artery resulted in a dose dependent reduction in forearm blood flow (panel **A**). Maximal reduction in forearm blood flow in response to SMTC was abolished in the presence of excess l-arginine but not d-arginine (panel **B**). Intraarterial infusion of the eNOS-agonist acetylcholine (ACh) resulted in a dose-dependent increase in forearm blood flow. This response was abolished in the presence of the nonselective NOS inhibitor l-NMMA but was unaffected by SMTC. *S*-methyl-l-thiocitrulline had no effect on NO-independent changes in blood flow in response to intraarterial sodium nitroprusside (SNP) (panel **C**) (adapted from Seddon et al. 2008. *Circulation* 117:1991-1996).

The first direct evidence for an involvement of nNOS-derived NO in the regulation of basal vascular tone in healthy humans has recently been provided by Seddon et al. (Seddon et al. 2008) in first-in-human studies with the nNOS-selective inhibitor SMTC. The local infusion of SMTC into the brachial artery of healthy subjects led to a significant dose-dependent reduction in basal forearm blood flow as measured by venous occlusion plethysmography. This response was abolished in the presence of excess l-arginine, but not d-arginine, indicating that the effects of SMTC were mediated through stereospecific inhibition of the l-arginine/NO pathway (Figure 1). The reduction in basal forearm blood flow induced by SMTC occurred at a significantly (>10-fold) lower concentration than l-NMMA, consistent with the greater potency of the former agent for nNOS inhibition. Importantly, SMTC had no effect on the increases in forearm blood flow elicited by intraarterial infusion of acetylcholine, which were inhibited by l-NMMA (Seddon et al. 2008) (Figure 1). Flow-induced vasodilatation, which is thought to be an eNOS-mediated response and is inhibited by l-NMMA, was also unaffected by SMTC (Seddon et al. 2009). Taken together, these results indicate that reduction in basal blood flow induced by SMTC results from selective nNOS inhibition, a mechanism that is independent of and is not required for eNOS-mediated responses to acetylcholine or increased flow.

**Figure 2:**
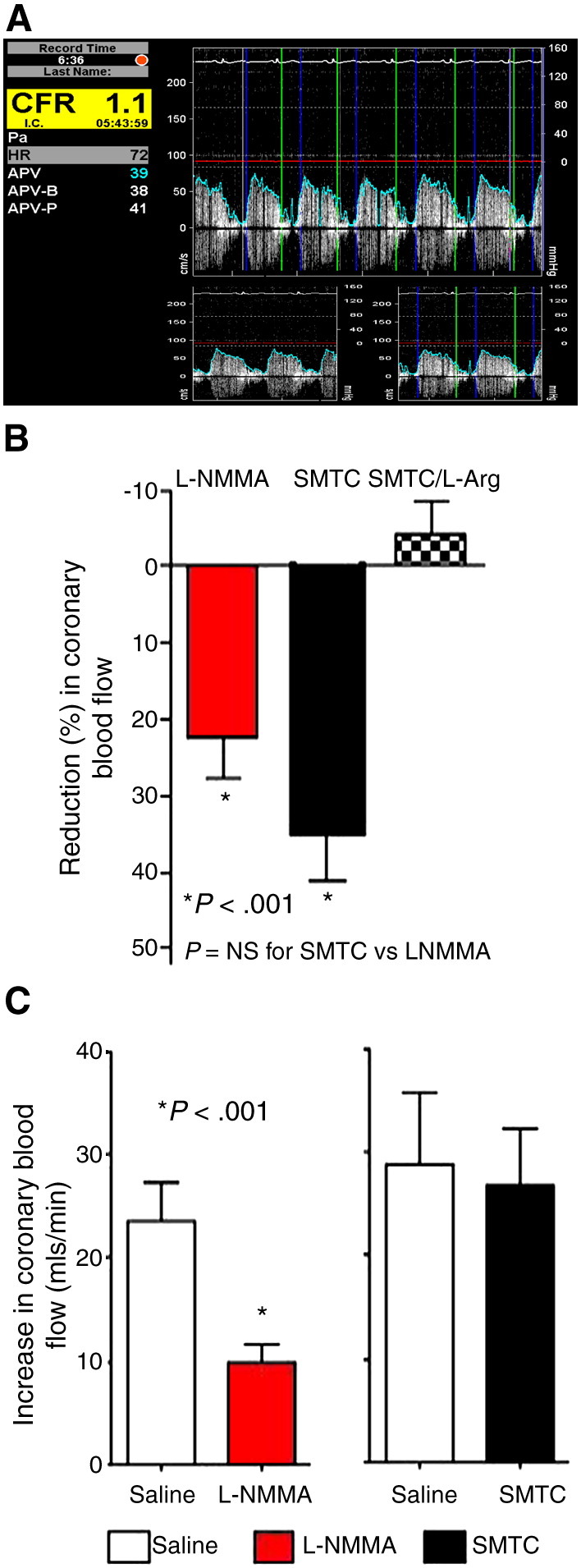
Effects of nNOS in the human coronary circulation. Intracoronary Doppler ultrasound traces demonstrating average peak velocity (APV) values at baseline and after infusion of the endothelial agonist substance P. Volumetric coronary blood flow can be obtained from the product of APV values and the cross-sectional area of the coronary vessel (calculated from measuring the diameter of the epicardial vessel with the use of quantitative coronary angiography [QCA]) at the time of APV recording (panel **A**). Doppler/QCA was used to derive changes in coronary blood flow in response to the nNOS-selective inhibitor SMTC and the nonselective NOS inhibitor l-NMMA during cardiac catheterization in patients who turned out to have unobstructed/smooth coronary vessels. As in the forearm, intracoronary infusion of the SMTC reduced coronary blood flow. This response was abolished in the presence of excess l-arginine but not d-arginine (panel **B**). Intracoronary infusion of the eNOS-agonist substance P increased coronary blood flow. This response was abolished in the presence of l-NMMA but was unaffected by SMTC (panel **C**) (adapted from Seddon et al. 2009. *Circulation* 119:2656-2662).

In further studies, Seddon et al. (Seddon et al. 2009) also investigated the potential role of local nNOS in the human coronary circulation. The effects of intracoronary infusion of SMTC were studied in patients undergoing cardiac catheterization who turned out to have angiographically normal coronary arteries. In these subjects, SMTC caused a significant reduction in basal blood flow as assessed by intracoronary Doppler and angiography. However, SMTC had no effect on the increases in flow elicited by intracoronary substance P infusion, which in contrast was inhibited by l-NMMA (Seddon et al. 2009) (Figure 2). These data indicate that the effects of SMTC on basal flow extend to the coronary circulation.

Previous studies with the use of l-NMMA have suggested that local NO is at least partly involved in mediating the peripheral and coronary vasodilator responses to mental stress, but the NOS isoform that is responsible has not been identified. With the use of a standardized mental stress protocol (the Stroop test), Seddon et al. (Seddon et al. 2008) found that the forearm vasodilator response was significantly attenuated by local forearm infusion of either SMTC or l-NMMA but not by a vasoconstrictor (norepinephrine) that reduced basal blood flow to a similar extent as the NOS inhibitors. These results strongly suggest a role for local nNOS-derived NO in regulation of mental stress-induced vasodilatation.

Kellogg et al. (2008) have recently provided further evidence for a functional association between axonal activity and nNOS-dependent regulation of vascular tone. In a series of studies in healthy volunteers, intradermal administration of the highly specific nNOS inhibitor 7-nitroindazole significantly attenuated increases in cutaneous blood flow in response to whole body heat, a process which involves centrally mediated axonal reflexes, but had no influence on blood flow changes in response to local application of heat, which, in contrast, involves locally mediated reflexes (Kellogg et al. 2008).

Taken together, the above data indicate that nNOS and eNOS have distinct local roles in the physiologic regulation of human coronary and peripheral microvascular tone in vivo and that these isoforms may therefore subserve distinct functions. Whereas eNOS-generated NO facilitates dynamic alterations in blood flow distribution (eg, in response to altered shear stress) and has antiatherosclerotic effects at the level of the endothelium, the tonic generation of NO by nNOS may be important for the regulation of basal vasomotor tone and blood flow.

## Current perspectives and future directions

**Figure 3:**
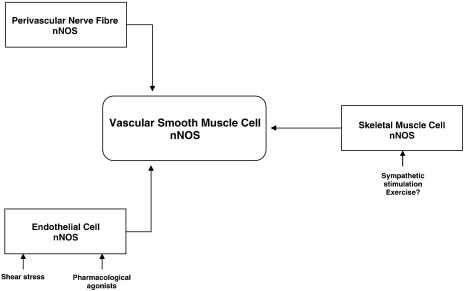
Potential in vivo sources of nNOS-derived NO. Schematic representation of the potential sources of nNOS and nNOS-derived NO in vivo. Neuronal NOS is known to be expressed in endothelial and smooth muscle cells within the vascular wall as well as skeletal muscle cells and perivascular nerve fibers. The nNOS-derived NO from one or more of these sources may influence vascular tone.

The conventionally accepted notion that tonic NO generation by endothelial eNOS regulates basal microvascular tone and blood flow is challenged by emerging data indicating that basal microvascular tone may be primarily regulated by local nNOS-derived NO (at least in the human forearm and coronary circulations), whereas eNOS may be responsible for changes in tone occurring in response to agonists or shear stress (Figure 3). The potentially independent regulation of basal flow versus stimulated endothelium-mediated increases in blood flow may account for the poor correlation between these two aspects of vascular function in clinical studies, especially in disease settings such as those that predispose to atherosclerosis where eNOS dysfunction may be more prominent.

Many fundamental questions regarding the local vascular influences of nNOS remain unanswered. The precise site and cellular source of nNOS that regulates microvascular tone remains to be defined. As outlined previously, evidence from experimental studies suggests that nNOS in the local vessel wall, perivascular nerves, and/or skeletal muscle could be involved (Bauser-Heaton and Bohlen 2007, Hatanaka et al. 2006, Thomas et al. 1998). However, it is not yet known which of these cellular sources is responsible for the effects of nNOS on basal microvascular tone in the human forearm and coronary circulation. Furthermore, different sources of nNOS may be involved in different beds or in different local vascular responses (such as regulation of basal tone, mental stress-induced increases in flow, or changes in blood flow during exercise).

The functional association between vascular nNOS and eNOS with respect to the regulation of tone and how this may be altered in disease settings also requires further study. Previous studies in animal models have suggested that there may be an inverse functional association between the two NOS isoforms within the vascular wall, with an up-regulation of nNOS when eNOS expression is reduced (Boulanger et al. 1998). How this may impact on the proposed distinct functions of eNOS and nNOS remains to be assessed. Another important issue is the potential impact of risk factors that lead to a reduction in NO bioavailability on the vascular actions of nNOS. It is well established that eNOS-mediated actions are impaired in the presence of hypercholesterolemia, diabetes, obesity, and systemic inflammation, and this is due at least in part to dysfunctional NOS activity secondary to oxidation of the critical cofactor tetrahydrobiopterin and/or direct NO scavenging by reactive oxygen species (Landmesser et al. 2004). What impact these conditions might have on nNOS-dependent regulation of vascular tone requires investigation. In this regard, it is intriguing that data from cardiac muscle suggest that dysfunctional nNOS signaling may itself result in increased reactive oxygen species generation (Eu et al. 2003, Kinugawa et al. 2005, Zhang et al. 2009).

Another important area that merits further study is the potential role of nNOS in the regulation of arterial blood pressure. Although microvascular tone would be expected to have a relatively limited impact on blood pressure, the fact that nNOS is also expressed in the central nervous system and the kidneys raises the possibility that its combined actions in these three systems may have significant effects on blood pressure regulation. The availability of a selective nNOS inhibitor validated for in vivo human use now paves the way for further direct investigation of some of these questions thereby to increase understanding of the roles of nNOS in human health and disease.
